# Acute Renal Failure in Critically Ill COVID-19 Patients With a Focus on the Role of Renal Replacement Therapy: A Review of What We Know So Far

**DOI:** 10.7759/cureus.8429

**Published:** 2020-06-03

**Authors:** Ahmad Raza, Adrian Estepa, Vincent Chan, Munnam S Jafar

**Affiliations:** 1 Internal Medicine, Abington Hospital-Jefferson Health, Abington, USA; 2 Internal Medicine, Abington Hospital, Jefferson Health, Abington, USA

**Keywords:** continuous renal replacement therapy (crrt), renal failure, severe acute respiratory syndrome coronavirus-2 (sars-cov-2), coronavirus disease 2019 (covid-19)

## Abstract

Acute renal failure remains a significant concern in all patients with the coronavirus disease 2019 (COVID-19) infection. Management is particularly challenging in critically ill patients requiring intensive care unit (ICU) level of care. Supportive care in the form of accurate volume correction and avoiding nephrotoxic agents are the chief cornerstone of the management of these patients. The pathophysiology of acute renal failure in COVID-19 is multifactorial, with significant contributions from excessive cytokine release. Gaining a better insight into the pathophysiology of renal failure will hopefully help develop more directed treatment options. A considerable number of these patients deteriorate despite adequate supportive care owing to the complexity of the disease and multi-organ involvement. Renal replacement therapy is used for a long time in critically ill septic patients who develop progressive renal failure despite adequate conservative support. Timing and choice of renal replacement therapy in critically ill COVID-19 patients remains an area of future research that may help decrease mortality in this patient population.

## Introduction and background

The global pandemic of novel coronavirus disease 2019 (COVID-19) caused by the severe acute respiratory syndrome coronavirus 2 (SARS-CoV-2) began in Wuhan, China in December 2019 and has since spread worldwide [1]. As of April 20, 2020, there have been more than 2.5 million reported cases and 169,000 deaths reported worldwide. SARS‐CoV‐2 is currently the world's most pressing public health threat and has a significant impact on the lives of people around the world. SARS‐CoV‐2 is an enveloped, positively charged, single‐stranded ribonucleic acid (RNA) virus belonging to the beta coronavirus genus. SARS‐CoV‐2 enters cells via the angiotensin-converting enzyme 2 (ACE2) receptor and is highly homologous to SARS‐CoV.

While SARS-CoV-2 primarily manifests as an acute respiratory illness with interstitial and alveolar pneumonia, it can affect multiple organs, such as the kidneys, heart, digestive tract, and nervous system. Kidneys are one of the primary and difficult to manage organs involved in critically ill patients with the COVID-19 infection. Renal failure is a well-established independent risk factor linked with increased in-hospital mortality in patients with severe sepsis regardless of etiology [2-3]. In previous reports of SARS and MERS-CoV infections, acute kidney injury (AKI) developed in 5% to 15% cases and carried a high (60% - 90%) mortality rate [4]. Early reports suggested a lower incidence (3% - 9%) of AKI in those with the COVID-19 infection [5-6]. Recently, a study involving 59 patients infected by SARS-CoV-2 found that 63% (32/51) of the patients exhibited proteinuria, 19% (11/59) of the patients had an elevated plasma creatinine level, and 27% (16/59) had an elevated urea nitrogen level [7].

Sepsis, a commonly encountered scenario in an intensive care unit (ICU), often leads to multi-organ dysfunction, and the kidney is one of the organs frequently affected. Acute kidney injury (AKI) occurs in about 19% of patients with moderate sepsis, 23% with severe sepsis, and 51% with a septic shock when blood cultures are positive [8]. According to a study published in 2007 after evaluating a varied population, sepsis was the most common cause of AKI in critically ill patients (47.5%) in 54 hospitals spread over 23 countries [9]. They inferred that oliguria was found to be more common in septic AKI (67% vs. 57%; P < 0.001), and septic AKI had a higher in-hospital mortality rate compared with non-septic AKI (70.2 vs. 51.8%; p < 0.001). The median duration of the ICU and hospital stay for survivors was longer for septic AKI (37 vs. 21 days; P < 0.0001).

Over the years, significant progress has been made towards learning how to detect AKI early, agreeing on an international consensus definition, delineating the pathophysiologic mechanisms which predispose to a high incidence of AKI in sepsis, trying to deduce logical protective and preventive strategies, and finally, on how to deliver the optimal renal support when the kidney fails. Renal replacement therapy has been the cornerstone of acute persistent or worsening renal failure in critically ill patients. We review the pathogenesis of acute renal failure in COVID-19 patients and discuss the role of renal replacement therapy in the management of these critically ill patients.

## Review

Pathophysiology of renal failure in COVID-19 patients

From our background knowledge of sepsis-induced AKI, we know that the exact pathophysiology of this illness is not known. However, it is generally accepted that it has a multi-pronged injury pathway. This form of AKI has components of ischemia-reperfusion injury, direct inflammatory injury, coagulation and endothelial cell dysfunction, and apoptosis [10-11].

SARS-CoV-2, a single-stranded RNA-enveloped virus, targets cells through the viral structural spike (S) protein that binds to the angiotensin-converting enzyme 2 (ACE2) receptor. Following receptor binding, the virus particle uses host cell receptors and endosomes to enter cells. A host type 2 transmembrane serine protease, TMPRSS2, facilitates cell entry via the S protein [12]. Once inside the cell, viral polyproteins are synthesized that encode for the replicase-transcriptase complex. The virus then synthesizes RNA via its RNA-dependent RNA polymerase. Structural proteins are synthesized, leading to the completion of assembly and release of viral particles [13-14].

So far, the majority of AKI in patients with COVID-19 seems to be acute tubular injury (ATI) in the setting of multiorgan failure and shock. In terms of histopathological findings, more tissue data are becoming available. A postmortem case series study reported that nine out of 26 autopsied COVID-19 patients in China experienced AKI, primarily characterized by light microscopy by diffuse proximal tubule injury and some frank necrosis [15]. In three cases, pigmented casts were found with high levels of creatine phosphokinase, possibly representing rhabdomyolysis. Distal tubules and collecting ducts showed only occasional cellular swelling and edematous expansion of interstitial space without significant inflammation. Lymphocyte infiltrates present in areas of nonspecific fibrosis, including subcapsular areas. The glomerular injury does not seem to be the predominant cause of worsening renal failure, though a variable degree of glomerular insult is described in different studies with one case report demonstrating collapsing glomerulopathy in patients with active COVID-19 infection [16].

One of the burning questions regarding acute renal failure in COVID-19 patients is whether COVID-19 directly infects the kidneys and causes renal failure. It starts with the entry of the virus into renal cells. The virus binds with the ACE2 receptor, which is well-expressed in the kidneys (particularly, podocytes and proximal tubular epithelial cells) and could serve as a binding site and mechanism of injury. One possibility to consider is that preexisting AKI induced by severe sepsis may be exacerbated by SARS-CoV-2. Once the initial injury occurs and alters the integrity of cell-cell junctions, the virus might then gain access to ACE2 on the luminal surface. ACE2 is expressed on the apical membrane, and it is unclear if the virus is capable of gaining entry to the luminal compartment. Coronavirus entry into host target cells also requires the fusion of the viral envelope with cellular membranes. Fusion-activates SARS-CoV peptides are created by specific proteolytic cleavage of the S proteins in a step called “priming.” Therefore, cell infectivity not only depends on ACE2 expression but is also governed by types of proteases found in a given cell type. In the kidney, detectable levels of the TMPRSS2 (transcript mouse, which the SARS-CoV-2 S protein, are only detectable in the proximal tubule S3 segment. Even then, TMPRSS2 is expressed at a very low level. It remains to be determined if other TMPSS or other proteases in the proximal tubule can mediate the priming of SARS-CoV-2 [17]. There are mixed reports regarding virus isolation from urine specimens of infected patients [18-19]. There are reports that SARS-CoV-2 has been isolated from the urine, but the presence of viral RNA has not been established as a link to kidney injury [20-21].

Electron microscope data from autopsy specimens have demonstrated spherical virus particles characteristic of coronavirus in proximal tubular epithelium [15]. The diameter of the virus particles and the length of spikes were like previously identified coronaviruses causing SARS and the Middle East respiratory syndrome (MERS). Furthermore, virus particles were clearly identified in podocytes, associated with foot process defacement, and occasional vacuolation and detachment of podocytes from the glomerular basement membrane. Virus infection was confirmed by immunofluorescence (IF) staining using an antibody targeting SARS-CoV nucleoprotein shared between β-coronaviruses. These findings indicate that the SARS-CoV-2 virus can directly infect the renal tubular epithelium and podocytes, which was associated with AKI and proteinuria in these patients with COVID-19.

The pathogenesis of kidney injury in COVID-19-infected patients is most likely to be multifactorial. In addition to the direct virulence of SARS-CoV-2, other secondary insults, especially renal medullary hypoxia, cytokine storms, organ cross-talk, hypo/hypervolemia, secondary infection with bacteria, other viruses, fungi, and drug-associated nephrotoxicity, can all contribute to AKI.

Renal replacement therapy in critically ill sepsis patients before COVID-19

Patients with septic shock comprise approximately 15% of ICU admissions and it is one of the leading causes of death in the ICU. Acute renal failure is a well-known complication in critically ill patients associated with increased rates of morbidity and mortality [22]. It is widely accepted that in such critically ill patients with acute renal failure, renal replacement therapy (RRT) may produce beneficial results by focusing on averting life-threatening derangements, such as reducing hypervolemia, removal of toxins/electrolytes, correcting acidosis that is associated with sepsis, and prevention of other complications associated with renal failure. The theory is that attenuating kidney-specific injury, and subsequently, non-kidney organ injury, may translate into overall improved survival and earlier improved kidney function. Proinflammatory and anti-inflammatory response leads to cellular and organ injury, most notably tumor necrosis factor (TNF), interleukin (IL)‐6, IL‐8, and IL‐10 [23]. Continuous renal replacement therapy (CRRT) has commonly used three types of depurative mechanisms: convection, diffusion, and adsorption by the filtering membrane. In addition to the above-mentioned functions, there is the possibility that CRRT may remove bacterial lipopolysaccharides and other inflammatory mediators [23]. This has led to the many studies that have been conducted over the past decade regarding the efficacy and appropriate timing for initiating RRT [24-25].

One of the first systematic reviews to explore whether the timing of RRT initiation had an impact on survival and kidney recovery in critically ill patients was published in 2011 [26]. Karvellas et al. conducted a meta-analysis that identified 15 unique studies (two randomized, four prospective cohorts, and nine retrospective cohorts) with the aim to investigate the impact of early versus late initiation of RRT. The analysis concluded that early RRT initiation was associated with reduced mortality compared to late initiation. Secondary outcomes suggested that early RRT could result in a higher rate of kidney recovery; however, no analysis was performed if RRT improved the length of stay in the ICU. Despite this being a strong study, limitations included significant statistical heterogeneity and publication bias towards smaller studies where early RRT was associated with a survival benefit [26].

In 2016, the Artificial Kidney Initiation in Kidney Injury (AKIKI) trial was published [27]. This was a large multicenter randomized trial consisting of 620 patients. Patients eligible included those who were admitted to an ICU with acute renal failure secondary to acute tubular necrosis due to ischemic or toxic injury and required mechanical ventilation, catecholamine infusion, or both. Patients with severe metabolic derangements (blood urea nitrogen (BUN) > 112 mg/dL, serum potassium (K) > 6 mmol/L, pH < 7.15, acute pulmonary edema due to fluid overload resulting in severe hypoxemia, etc.) were excluded. Patients were then assigned to an early-strategy group (RRT initiated within six hours of documentation of acute kidney injury) and a delayed strategy group (RRT initiated if one of the above abnormalities developed or oliguria/anuria lasted for > 72 hours). Statistical analysis showed that, for the primary outcome of mortality, there was no significant difference between the two groups. Furthermore, among the secondary outcomes, catheter-related bloodstream infections were significantly higher in the early RRT group. Significant limitations of this study included the questionable power of the study and limitation to patients with advanced acute kidney injury Kidney Disease Improving Global Outcomes (KDIGO Stage 3) [27].

In contrast, the Early Versus Late Initiation of Renal Replacement Therapy in Critically Ill Patients with Acute Kidney Injury (ELAIN) trial was also published in 2016, which had different results from that of the AKIKI trial [24]. The ELAIN trial was a single-center randomized trial that consisted of 231 patients. Like the AKIKI trial, the aim again was to focus on the optimal timing of RRT initiation in critically ill patients. The main differences in inclusion criteria were that the ELAIN trial included KDIGO Stage 2 or greater patients who were assigned to early RRT (within eight hours of Stage 2 AKI diagnosis) or delayed RRT (initiated within 12 hours of Stage 3 AKI) or any absolute indications for RRT, such as blood urea nitrogen (BUN) > 100 mg/dL, K > 6 mEq/L, urine output (UO) < 200 mL/12 hours or anuria). For this trial’s primary outcome of mortality, this study was able to conclude that early initiation of RRT significantly reduced the 90-day mortality rate compared with delayed initiation of RRT. Moreover, with regard to secondary outcomes, early initiation of RRT showed a statistical significance in reducing the duration of RRT needed, enhanced recovery of renal function, reduced duration of mechanical ventilation, and decreased length of stay. Limitations of this study included that this was a single-center trial and limited generalizability due to all patients being surgical patients.

Most recently, in 2018, another multicentered, randomized, controlled trial was published studying septic shock patients with severe acute kidney injury [28]. However, these patients were without life-threatening complications due to acute kidney injury. A total of 488 patients underwent randomization to two groups - those receiving RRT within 12 hours of documented acute kidney injury versus a delay of 48 hours if renal recovery had not occurred. Similar to the AKIKI trial, this study concluded that among patients with septic shock who had severe acute kidney injury, there was no significant difference in the overall 90-day mortality rate in early initiation compared to delayed initiation. Furthermore, no significant difference was found with regards to days free of mechanical ventilation, vasopressor use days, and length of stay in the ICU and hospital [28].

So, while it is undeniable that acute kidney injury is a frequent complication of patients hospitalized in the ICU with septic shock, managing this kidney injury and whether to provide RRT/when to initiate remains unclear. While it is widely accepted that RRT should be initiated in life-threatening complications of acute kidney injury, such as hyperkalemia, metabolic acidosis, volume overload causing hypoxemia, etc., data regarding initiation of RRT without said complications remains mixed. 

Renal failure in critically ill COVID-19 patients

Throughout the course of the pandemic, there has been growing evidence that suggests that patients with severe COVID-19 infection may have a cytokine storm syndrome [1]. Cytokine storm syndrome is among the many other possible causes of acute renal failure in these critically ill patients. While the mainstay of management of COVID-19 infection is supportive with the leading cause of mortality resulting from acute respiratory distress syndrome (ARDS), multi-organ failure in these patients is possibly associated with a hyperinflammatory syndrome characterized by hypercytokinemia. Increased levels of IL-2, IL-6, IL-7, IL-8, IL-10, granulocyte-colony stimulating factor (G-CSF), interferon (INF)-gamma, and TNF have all been identified [2]. Because high viral titer, along with a subsequent robust inflammatory cytokine response, has been associated with higher morbidity and mortality, treatments that counteract these cytokines has been a hotbed for research and clinical trials. 

Therapies, such as INF-gamma inhibitors, corticosteroids, intravenous immunoglobulin G (IVIG), TNF blockers, chloroquine, and others, have been postulated to be beneficial in the treatment of cytokine storm; however, they have not yet shown significant efficacy. One of the treatment modalities that remains controversial in the treatment of sepsis syndrome is the use of CRRT. As previously mentioned, CRRT can remove inflammatory factors, thus blocking the cytokine storm syndrome, and, therefore, ultimately reducing the damage done to multiple organs [3].

Over the past three decades, the use of various forms of continuous and prolonged intermittent RRT (PIRRT) in the management of critically ill patients with AKI has increased dramatically. Comparing outcomes between the modalities is complicated. Several randomized controlled trials comparing intermittent to continuous RRT have been performed, although many of these trials have been hampered by issues of patient selection and protocol adherence, excluding patients, or having them cross between treatment arms because of hemodynamic instability [29-31]. The largest of these trials, the Hemodiafe study, enrolled 360 patients across 21 ICUs in France. No difference in survival at two, 60, or 90 days (60-day survival at 31.5% with ischemic heart disease (IHD) vs. 32.6% with CRRT, p = 0.98), or recovery of kidney function was observed between the groups. It should be noted, however, that the median treatment duration for each intermittent hemodialysis session was 5.2 hours, significantly longer than is typical in clinical practice [32]. Three systematic reviews and meta-analyses of the modality of renal support in AKI have been published in the last five years, all of which found no differences in mortality or recovery of kidney function across modalities [33-35].

Multiple studies have been done to date regarding the efficacy of the removal of these cytokines. Like the trials evaluating the effectiveness of CRRT on mortality of patients with sepsis syndrome, these studies have been met with mixed results. In a 2019 study, Zuccari et al. performed a prospective observational study on critically ill patients with sepsis/septic shock who underwent RRT and hemoadsorption with Cytosorb [36]. Plasma levels of TNF, IL-1, 6, 8, and 10 were measured at baseline, six hours, and 24 hours. Only plasma levels of IL-8 were found to have a significant reduction. Kellum et al. sought to compare continuous venovenous hemofiltration (CVVH) to continuous venovenous hemodialysis (CVVHD) in terms of removal of inflammatory mediators TNF-alpha, IL-6, 10, and soluble L-selectin (sL-selectin) [37]. This study concluded that CVVH was superior to the removal of TNF-alpha compared to that of CVVHD; however, the mean plasma concentrations of IL-6, 10, and sL-selectin were unchanged over time and between the two therapies.

Currently, the Tocilizumab vs. CRRT in Management of Cytokine Release Syndrome (CRS) in COVID-19 (TACOS) clinical trial is underway in China, comparing tocilizumab to CRRT in the treatment of the cytokine storm in COVID-19 patients. Tocilizumab was approved in 2017 to treat cytokine release syndrome (CRS), a form of cytokine storm caused by chimeric antigen receptor T (CAR-T) treatment in cancer patients. The primary outcome of this trial will be normalization of fever and oxygen saturation by Day 14 with notable secondary outcomes, including the length of hospitalization, time to negative corona viral reverse transcription-polymerase chain reaction (RT-PCR), all-cause mortality, and change of baseline cytokines (IL-1, 6, 8, 10, TNF-alpha) [38].

A final issue related to modality of therapy is the relative benefit of convective (hemofiltration) versus diffusive (hemodialysis) therapies. Convective therapies are generally thought to provide better clearance of solutes with molecular weights greater than 1,000 Daltons [39-40]. It has, therefore, been suggested that convective therapies might provide an added benefit in patients with sepsis-associated AKI through enhanced removal of proinflammatory mediators [41]. However, the cytokine clearances attainable with even high-volume CVVH are trivial in comparison to endogenous production, and cytokine removal by hemofiltration is non-selective, resulting in the removal of both pro- and anti-inflammatory mediators [42]. In addition, the effects of convective solute flux as the result of internal filtration/back-filtration and protein concentration polarization, along the membrane surface, when high-flux membranes are employed may minimize the differences in solute clearance between convective and diffusive therapies [43]. A systemic review of randomized trials published in 2014 focused on convective versus diffusive dialysis strategies in patients with chronic kidney disease (CKD) and found that convective dialysis may reduce cardiovascular but not all-cause mortality, and effects on nonfatal cardiovascular events and hospitalization were inconclusive [44]. 

## Conclusions

Understanding the pathophysiology of acute renal failure in COVID-19 patients is an actively evolving issue. In these critically ill patients, it is essential to prevent and actively manage acute renal failure to decrease mortality. It would be exciting to see the comparisons between different dialysis techniques to help manage the cytokine storm. There have been discussions regarding the superiority of some dialysis techniques in the management of COVID-19 infected patients and whether one method is superior to others. To date, no data is available to compare different modalities in COVID-19 patients, and it remains an area of future research.
